# Cultivating crayfish (*Procambarus clarkii*) significantly enhances the quantity and diversity of soil microorganisms: evidence from the comparison of rice-wheat and rice-crayfish rotation models

**DOI:** 10.3389/fmicb.2025.1528883

**Published:** 2025-02-03

**Authors:** Hui Xu, Dan Wang, Xuguang Li, Jiajia Li, Yu Xu, Zhiqiang Xu

**Affiliations:** ^1^Freshwater Fisheries Research Institute of Jiangsu Province, Jiangsu Freshwater Fisheries Research Institute, Nanjing, China; ^2^Key Laboratory of Genetic Breeding and Cultivation for Freshwater Crustacean, Ministry of Agriculture and Rural Affairs, Freshwater Fisheries Research Institute of Jiangsu Province, Nanjing, China; ^3^College of Marine Science and Fisheries, Jiangsu Ocean University, Lianyungang, China

**Keywords:** rice-crayfish rotation, rice-wheat rotation, integrated rice farming, *Procambarus clarkii*, soil microorganisms

## Abstract

Integrated farming of rice (IFA), as a time-honored agricultural model, can effectively increase agricultural productivity and provide ecological benefits. Rice-wheat rotation and rice-crayfish (*Procambarus clarkii*) rotation are two most widely applied IFA patterns in China. In this study, we compared the differences in soil microbial communities and predicted their functions in these two IFA models by sequencing the 16s rRNA and analyzing the bioinformation. The results showed that crayfish farming effectively increased the abundance and diversity of soil microorganisms. The main differentially abundant phyla between the two groups were Actinobacteriota, Bacteroidota, and Desulfobacterota, while the main differentially abundant genera were *Bacteroidetes_vadinHA17*, *Sphingomonas*, and *Thiobacillus*. The Similarity Percentages (SIMPER) analysis indicated that these species also had the highest contribution to the differences in microbial composition between the two groups. Random forest prediction analysis was employed to identify potential biomarkers to distinguish the two microbial communities. Actinobacteriota, Desulfobacterota, and Spirochaetota were identified as potential biomarker phyla. *Streptomyces*, *Kribbella*, and *Paludibacter* could serve as potential biomarker genera. Functional Annotation of Prokaryotic Taxa (FAPROTAX) analysis revealed that the dominant bacterial functions in the rice-wheat rotation model were aerobic chemoheterotrophy and chemoheterotrophy. In contrast, the bacterial functions in the rice-crayfish rotation model were more diverse, primarily including methylotrophy, human pathogens all and methanotrophy. The results of co-occurrence network analysis showed that crayfish farming enhanced the modularity of the soil microbial community, and revealed that the microbial network in rice-wheat soil had fewer nodes and more edges, which implying more internal connections. In conclusion, the wheat planting and crayfish farming drove significant differences in the soil microbial communities of paddy fields, with Actinobacteriota and Desulfobacterota identified as potential biomarkers. Compared to wheat cultivation, the rotation system incorporating crayfish farming enhanced the richness and diversity of soil microbial species and functions, increased the modularity of the microbial community, and promoted the presence of keystone species with connecting roles. Our study would not only clarify the effects of different IFA models on soil microbial communities, and should also provide valuable insights for future adjusting cropping patterns and controlling current soil microbial ecological problems.

## 1 Introduction

Integrated rice farming involves the introduction of other species into rice paddies to fully utilize land, water, and other environmental resources (Li et al., 2018; Hu et al., 2021). It further leverages the interactions between organisms to construct ecological networks, enabling the circulation of nutrients within the rice paddies and reducing the need for additional feed inputs, thereby promoting sustainable agriculture (Bashir et al., 2020). Integrated farming of rice and aquaculture animals (IFRAA) combines rice cultivation with aquaculture, such as ducks, fish, crabs, and turtles (Hu et al., 2013; Zhang et al., 2016; Yang et al., 2018; Zhang et al., 2023). The traditional rice-fish co-cultivation system has a history of over 1,200 years (Ge et al., 2023). IFRAA is considered to have the potential to enhance land and water resource utilization, obtain more agricultural products, and reduce greenhouse gas emissions (Bashir et al., 2020). Implementing IFRAA could be an agricultural model that improves production performance and ecological benefits (Fang et al., 2023; Liu et al., 2024), marking an important direction for the development of modern intensive agriculture (Zhu et al., 2022).

Among several IFRAA models, the rice-crayfish (*Procambarus clarkii*) co-cultivation model yields the highest economic benefits (Xu et al., 2022). This farming model originated in Louisiana, United States (Si et al., 2017). In recent years, this model has rapidly developed and been widely applied in China (Ge et al., 2023), becoming the largest in terms of area among China’s IFRAA models (Xu et al., 2022). Research has already discovered that the integrated rice-crayfish farming model offers significant ecological benefits. The residual feed and feces produced by crayfish may serve as excellent sources of biological fertilizers, helping to increase soil nutrients such as nitrogen, phosphorus, and potassium (Wang et al., 2023). The burrowing behavior of crayfish can enhance vertical material exchange (Si et al., 2018), increasing the organic carbon, nitrogen, and potassium content in soil at depths below 20 cm, reducing soil bulk density, improving soil properties, and increasing the biomass and depth of rice roots (Si et al., 2017). Additionally, the integrated rice-crayfish farming model contributes to enhancing soil microbial diversity and accumulation of biomass (Li et al., 2022).

The internal factors of the integrated rice-crayfish farming model are intricately connected and facilitated by ecological networks (Xu et al., 2022). Microorganisms, being the most widespread, active, and sensitive components in soil, possess diverse genetic and metabolic information. Consequently, they can effectively reflect environmental changes. Soil microorganisms play a crucial role in the transfer of matter and energy within the geochemical cycles (Mendes et al., 2013) and are often used as key indicators to assess soil ecosystem health and soil quality. Studying the community structure of soil microorganisms helps in understanding material cycles and evaluating soil quality (Schofield et al., 2018). Numerous studies have already explored environmental microorganisms in integrated rice-crayfish farming models. It has been found that raising crayfish in rice paddies can enhance the alpha diversity of environmental microorganisms in the short term (Wang Y. et al., 2021), and stocking density can alter soil bacterial composition and diversity (Hou et al., 2024). However, compared to measuring microbial diversity, analyzing microbial co-occurrence networks provides a more comprehensive reflection of environmental changes on microorganisms (Prosser et al., 2007; Barberan et al., 2012). This is because microbial communities involve complex interactions, both direct and indirect, which are challenging to identify individually. Co-occurrence networks can integrate these interactions and identify and quantify them (Proulx et al., 2005; Karimi et al., 2017), thereby further uncovering the functions and ecological niches of uncultured microorganisms.

Rice-wheat rotation, as an essential method of grain production, has long been widely practiced in the southeastern regions of China (Zheng et al., 2000). Currently, most studies focus on comparing environmental microorganisms in rice monoculture and rice-crayfish co-culture systems, while exploration of comprehensive rice field utilization models such as rice-crayfish rotation and rice-wheat rotation remains limited. This study aimed to investigate the effects of planting wheat and farming crayfish during the rotation period on the composition and function of soil microbial communities. We hypothesized that planting wheat or raising crayfish during the rotation period could significantly alter the types and quantities of soil microorganisms, thereby changing their ecological functions, co-occurrence networks and impacting agricultural environments. To addressed this hypothesis, the soil samples from the rice-wheat rotation and rice-crayfish rotation models in Jiangsu Province, southeastern China were obtained, the microbial community structure and ecological network relationships in the soil were investigated, and the effects of different rotation patterns on soil microbial communities were evaluated.

## 2 Experimental methods

### 2.1 Field environment and sample collection

**Figure 1:**
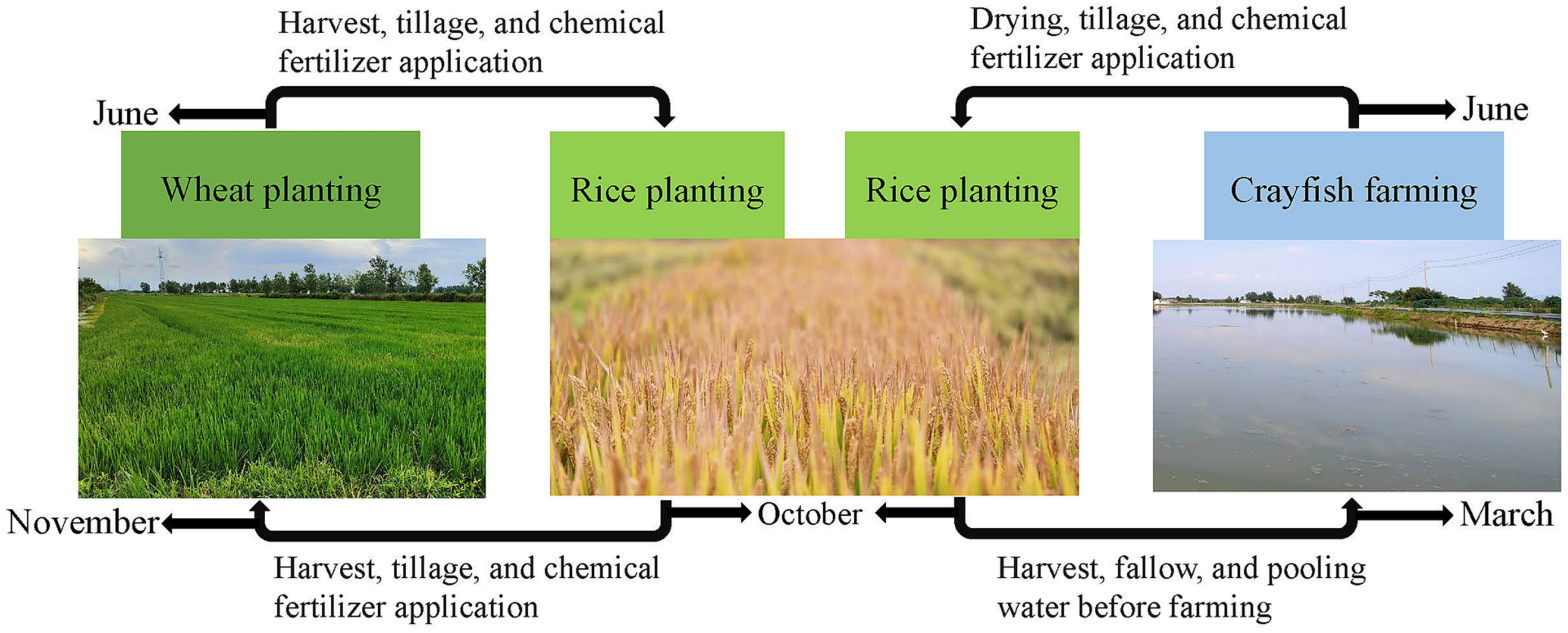
The farming practices for the rice-wheat and rice-crayfish rotation models.

The experimental site was located in Baoying County, Yangzhou City, Jiangsu Province (33°17′N, 119°36′E). This region has a subtropical monsoon climate with an annual average precipitation of 966 mm, 2,181 h of sunshine, and an annual average temperature of 14.4°C. The experiment was set up in two groups: the rice-wheat rotation model (group RW) and the rice-crayfish rotation model (group RC). Each group had three adjacent replicate plots. The rice cultivars were all Nanjing 9108. The farming practices for the rice-wheat rotation group were as follows (Figure 1): wheat was planted from November to June of the following year, and rice was planted from June to October. The fields were plowed and fertilized with chemical fertilizers after each harvest. The farming practices for the rice-crayfish rotation group were as follows (Figure 1): 300 kg/ha crayfish larvae were raised from March to June, the fields were dried after the crayfish farming, and chemical fertilizers were applied, with rice planted from June to October. The fertilizer application was the same as in the rice-wheat rotation model: 350 kg/ha chemical fertilizer (N: P: K, 17:13:15) were applied before rice planting. The same fertilizer application was token before wheat planting. Each plot had been applied the corresponding farming practices for more than 3 years.

Soil samples were collected on June 14, 2023. This time point was after the completion of the rotation in both models and before rice planting. Three points were taken using the diagonal method in each plot. Surface soil samples were collected within a 10 × 10 m range at each point, with a depth of 0–20 cm. Each group contained nine samples and a total of 18 samples were obtained. The obtained samples were transported to the laboratory on dry ice and soil DNA extraction was conducted as soon as possible.

### 2.2 DNA extraction and high-throughput sequencing

Total soil genomic DNA from samples was extracted with Power Soil DNA Isolation Kit (MoBio Laboratories, California, United States). DNA concentration was determined by Nanodrop 2000 spectrophotometer (Thermo, Massachusetts, United States). The purity and integrity were evaluated with 1% agarose gel electrophoresis. According to the concentration, DNA was diluted to 1 ng/μL using sterile water. The V3-V4 region of 16S rRNA genes were amplified with universal primer set 338F (5′-ACTCCTACGGGAGGCAGCAG-3′) and 806R (5′-GGACTACHVGGGTWTCTAAT-3′). All PCR reactions were carried out in 30 μL reactions with 15 μL of High-Fidelity PCR Master Mix (New England Biolabs, Massachusetts, United States); 0.2 μM of forward and reverse primers, and about 10 ng template DNA. The thermocycling conditions were as follows: 98°C pre-degeneration for 1 min, denaturation at 98°C for 10 s, annealing at 50°C for 30 s, and extension at 72°C for 30 s, with a total of 30 cycles, followed by a final elongation step at 72°C for 5 min. At last, the library was sequenced on the Illumina MiSeq platform (Illumina, California, United States). The raw sequence data were deposited into the National Center for Biotechnology Information (NCBI) database (PRJNA1185463).

### 2.3 Bioinformation analysis

The raw reads were spliced and filtered to obtain clean data. Microbiome bioinformatics were performed with QIIME 2 following the official tutorials. The raw reads were normalized, with the threshold set at 95% of the minimum sample sequence quantity. The average sequencing depth was 50,971 and the average number of amplicon sequence variants (ASV) was 29,753 (Supplementary Table S1). Alpha diversity indices (ACE, Chao 1, Shannon, Simpson) were calculated to analyzed the abundance and diversity of microbial communities. Venn, Non-Metric Multi-Dimensional Scaling (NMDS), Adonis analysis and Unweighted Pair Group Method with Arithmetic Mean Analysis (UPGMA) were performed based on the Bray–Curtis distance to explore the difference between models. Species annotation was performed using Quantitative Insights Into Microbial Ecology 2 (QIIME2) software based on Silva Database^1^ at the 95% similarity level. Significant differentially abundant species were identified using the Mann–Whitney test. Species with contribution to the difference between groups were annotated with SIMPER (Similarity percentage) analysis. Potential biomarkers were screened using Random Forests (RF) prediction analysis. Functional annotation was performed with the Functional Annotation of Prokaryotic Taxa (FAPROTAX) database. Group differences in functions were analyzed using Partial Least Squares Discriminant Analysis (PLS-DA). These analyses were implemented in R environment (version 4.4.2) with the following R packages: vegan, ape, ggplot2, igraph, psych. Significant differentially abundant functions were identified with the STAMP software (version 2.1.3).

### 2.4 Co-occurrence network analysis

The co-occurrence network analysis was conducted using R packages (Hmisc, igraph). A matrix was constructed by species with frequencies higher than 50%. Only ASVs with correlation coefficients |*r*| > 0.8 and adjusted *p* < 0.05 were retained based on spearman correlation analysis. The topological properties of the nodes were calculated, including *Zi* values and *Pi* values. *Zi* represents the z-scores of the within-module connectivity for node *i*, and *Pi* is the participation coefficient calculated based on the between-module connectivity for node *i*. Nodes were categorized into four types based on their values: peripherals (*Zi* ≤ 2.5, *Pi* ≤ 0.62), connectors (*Zi* ≤ 2.5, *Pi* > 0.62), module hubs (*Zi* > 2.5, *Pi* ≤ 0.62), and network hubs (*Zi* > 2.5, *Pi* > 0.62). The analysis results were visualized using Gephi software (version 0.10.0), with nodes colored according to different modules. The topological properties of the network were calculated.

## 3 Results

### 3.1 Sequencing data processing and community diversity analysis

**Figure 2:**
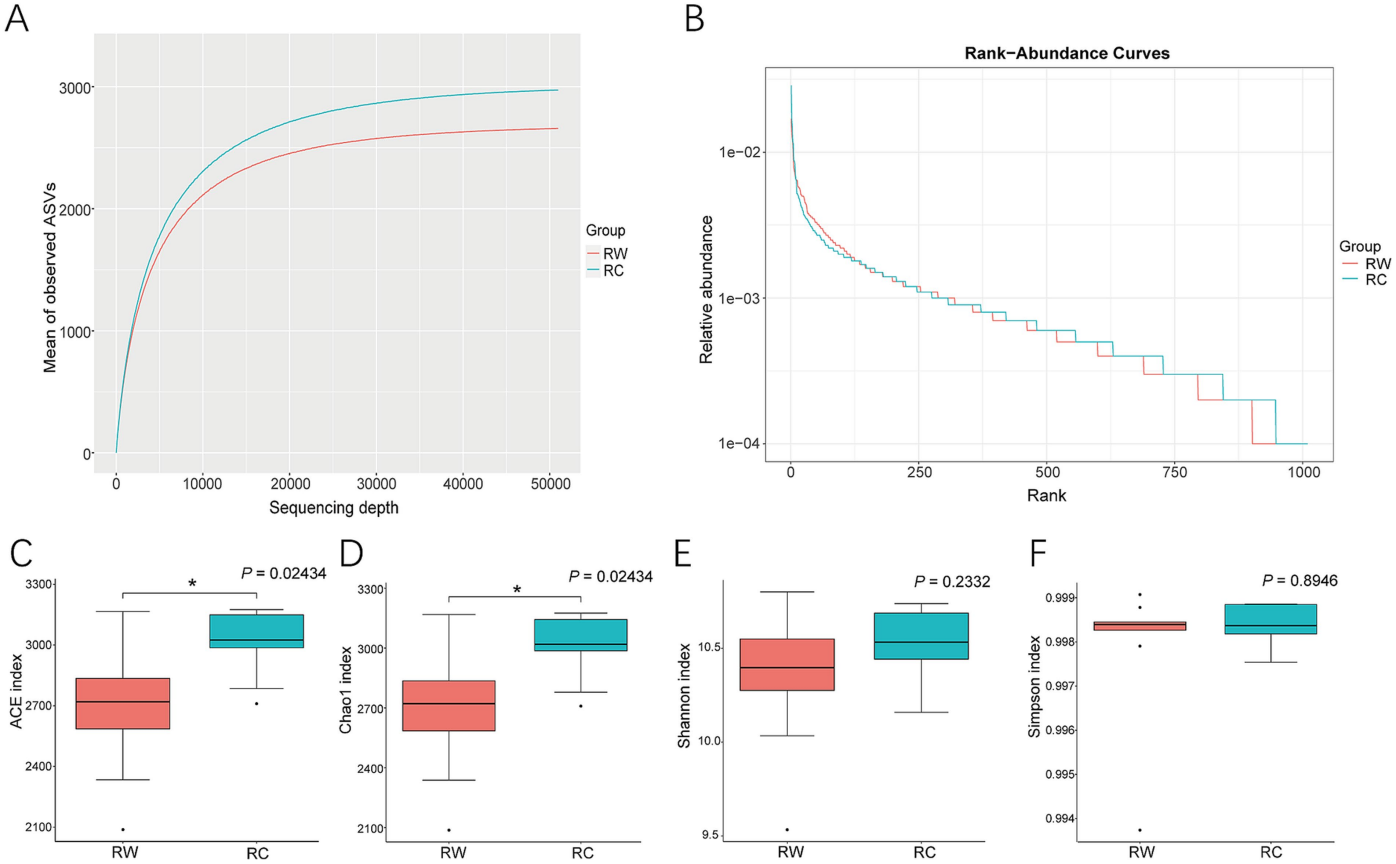
Alpha diversity analysis of soil samples. **(A)** Rarefaction Curve for two groups. **(B)** Rank Abundance Curve for two groups. **(C)** Analysis of ACE index. **(D)** Analysis of Chao1 index. **(E)** Analysis of Shannon index. **(F)** Analysis of Simpson index. Group RW represents soil after wheat planting and group RC represents soil after crayfish farming. Differences between samples were analyzed using the Kruskal–Wallis method and indicated in the figure. “*” indicates significant differences between groups (*p* < 0.05).

Compared to rice-wheat planting model, more diverse microorganisms were observed in soil when crayfish were raised (Figure 2). The rarefaction curves showed that more ASVs were observed in group RC with the same sequencing depth (Figure 2A). The rank abundance curves analysis revealed that group RC had a broader curve, indicating higher soil microbial richness (Figure 2B).

Calculations of microbial diversity indexes revealed that soil microbial communities in crayfish-raised plots had significant higher ACE and Chao1 indexes (*p* < 0.05). Compared to wheat planting, raising crayfish increased the ACE index by 12.06% and the Chao1 index by 11.84%, indicating that raising crayfish effectively enhances the number of soil microbial species (Figures 2C,D). There were no significant differences in Shannon and Simpson indexes between the two groups (*p* > 0.05, Figures 2E,F).

**Figure 3:**
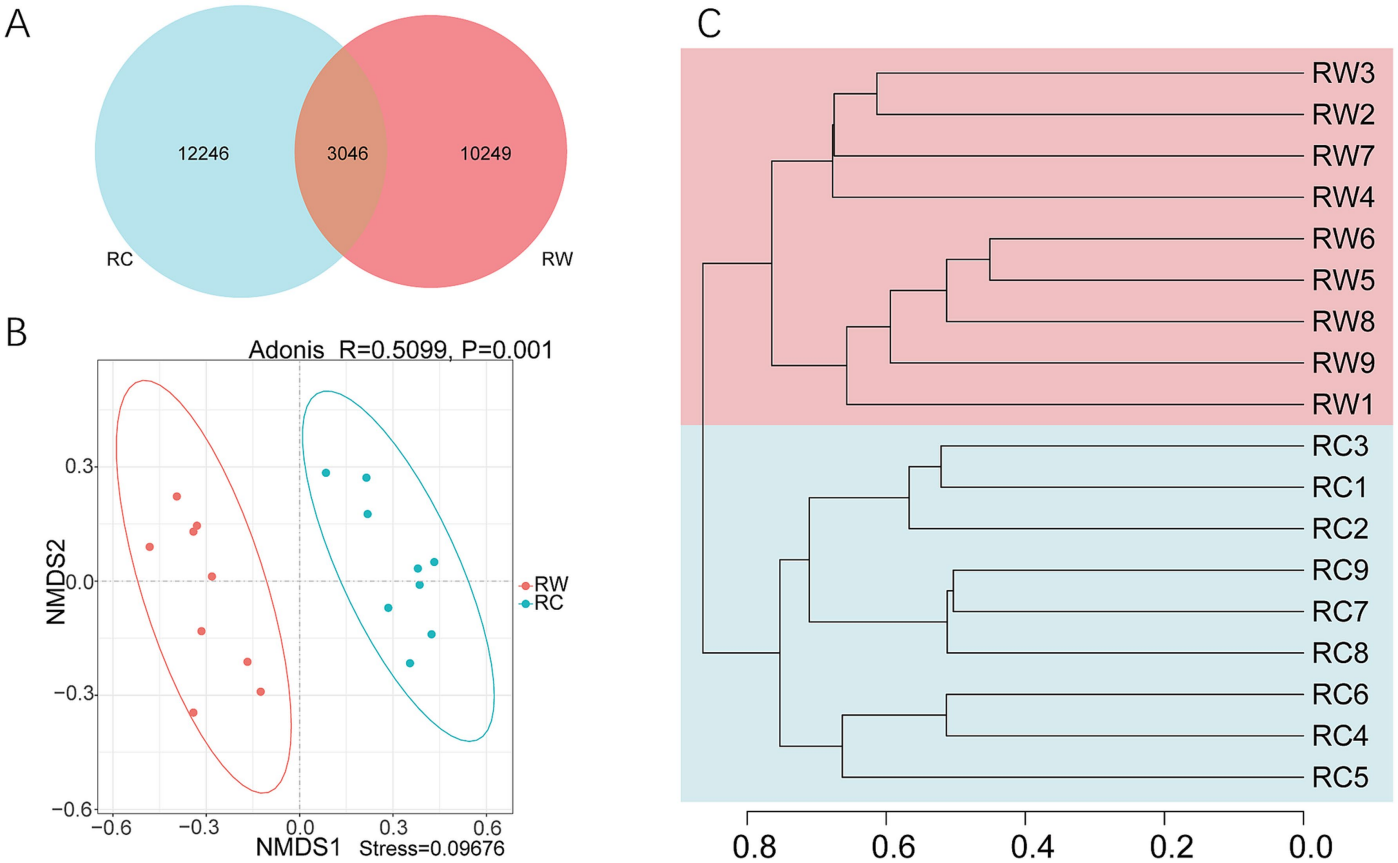
Beta diversity analysis of samples. **(A)** Venn diagram. **(B)** NMDS (Non-Metric Multi-Dimensional Scaling) and Adonis analysis. **(C)** UPGMA (Unweighted Pair-group Method with Arithmetic Mean) analysis. Group RW represents soil after wheat planting and group RC represents soil after crayfish farming. These analyses were based on Bray – Curtis dissimilarity.

The differences in soil microbial community composition between the two groups were further explored. A Venn diagram revealed that there were 3,046 shared ASVs between the two groups, but each group had more unique ASVs (Figure 3A). Group RC had more unique ASVs (totaling 12,246). The NMDS analysis established a reliable planar model (stress = 0.09676, Figure 3B). The analysis results showed that the two groups of samples were completely separated on axis 1. Adonis analysis indicated significant differences between the two groups (*p* = 0.001). The UPGMA showed that the samples clearly clustered into two branches on the dendrogram (Figure 3C). These findings suggest that the treatments of wheat planting and crayfish farming resulted in significant differences in soil bacterial communities.

### 3.2 Soil microbial composition and difference analysis

**Figure 4:**
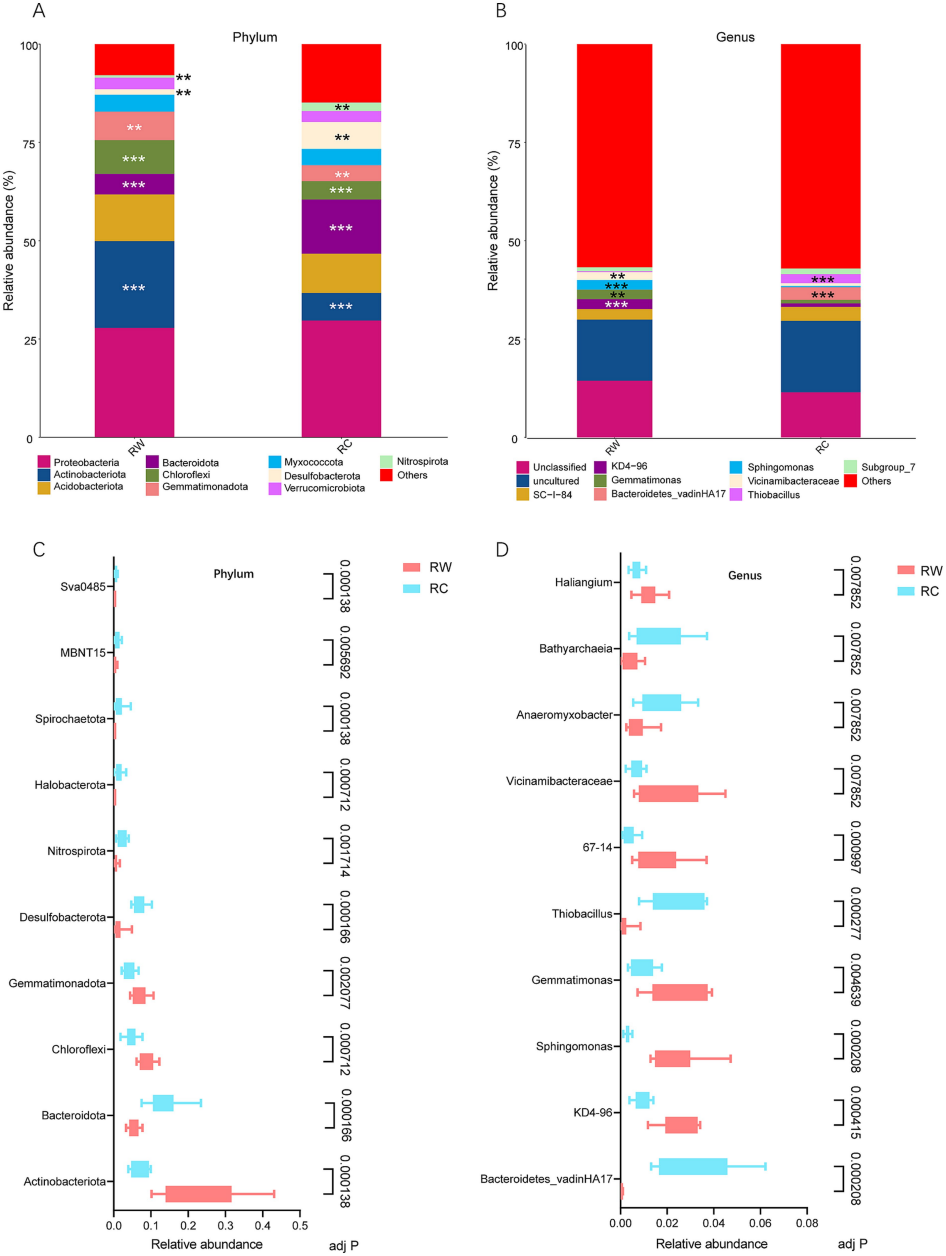
Bacterial community composition and differential species identified in the two rice farming models. **(A)** Bar chart of dominant species composition at the phylum level (top 10 with the highest relative abundance). **(B)** Bar chart of dominant species composition at the genus level (top 10 with the highest relative abundance). **(C)** Test results of the top 10 differential bacterial phyla by relative abundance. **(D)** Test results of the top 10 differential bacterial genera by relative abundance. Group RW represents soil after wheat planting and group RC represents soil after crayfish farming. Significance was tested using the Mann–Whitney test. “**” means *p* < 0.01 and “***” means *p* < 0.001. Adj *P* means adjusted *p* value.

**Figure 5:**
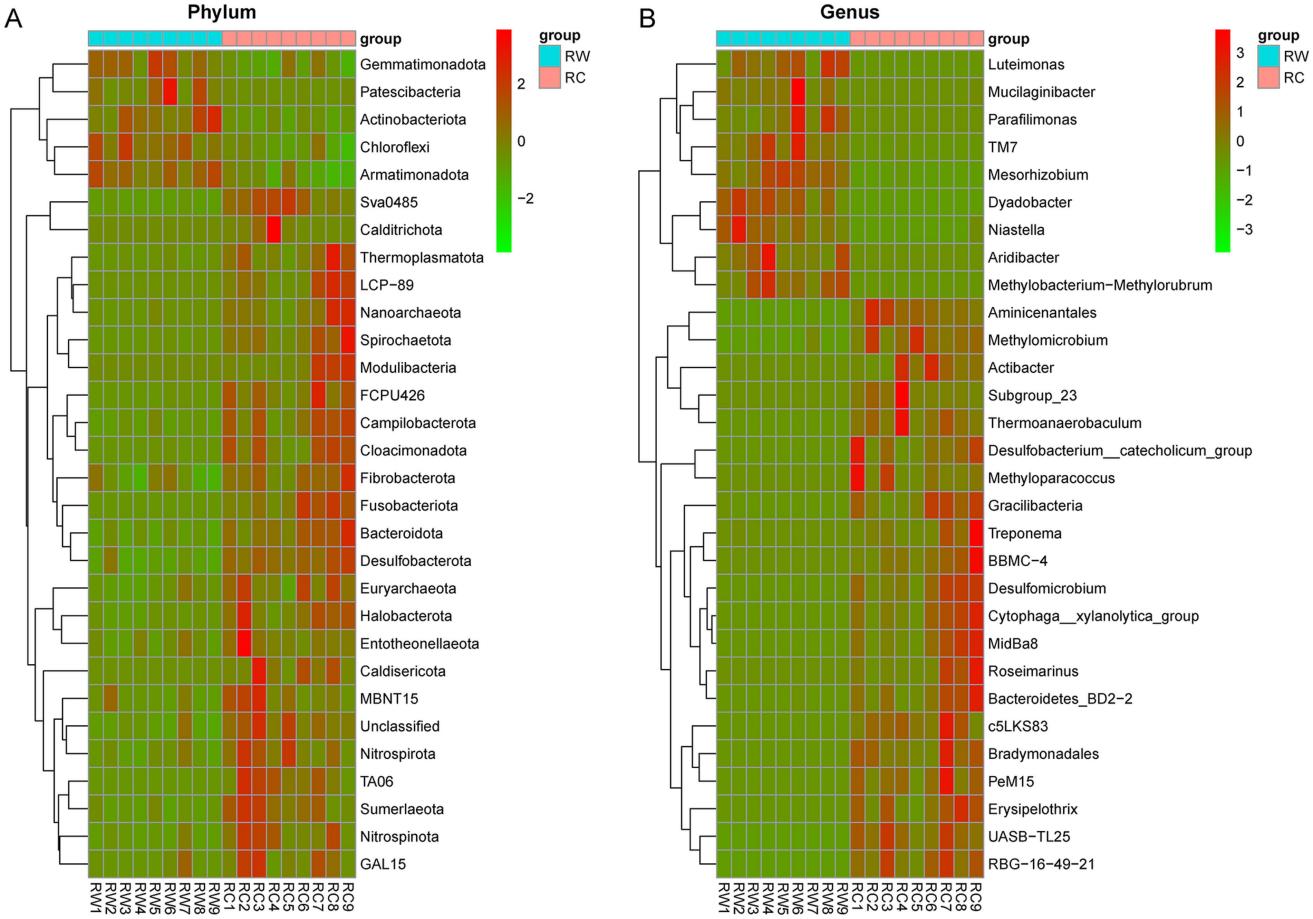
The clustering heatmap of the top 50 most abundant species with significant differences. **(A)** Heat map on phylum level. **(B)** Heat map on genus level. RW represents soil after wheat planting and group RC represents soil after crayfish farming. The red boxes mean positive relevance and green boxes mean negative relevance.

Based on species annotation results, the top 10 most abundant species at the phylum level in both groups were Proteobacteria, Actinobacteriota, Acidobacteriota, Bacteroidota, Chloroflexi, Gemmatimonadota, Myxococcota, Desulfobacterota, Verrucomicrobiota, and Nitrospirota (Figure 4A). At the genus level, eight genera were annotated: *SC-I-84*, *KD4-96*, *Gemmatimonas*, *Bacteroidetes_vadinHA17*, *Sphingomonas*, *Vicinamibacteraceae*, *Thiobacillus* and *Subgroup_7* (Figure 4B). The dominant phylum in both groups was Proteobacteria, with average relative abundances of 27.81 and 29.68%, respectively. The dominant genus was *SC-I-84*, with average relative abundances of 2.69 and 3.53%, respectively. The relative abundances of species were tested using the Kruskal-Wallis test. After standardization, Z-scores were obtained and a heatmap was created. The results showed that species were clearly divided into two clusters, corresponding to the wheat planting group (RW) and the crayfish farming group (RC) (Figures 5A,B). More microorganisms were enriched in the RC group.

To further explore the differential species between the two groups, the relative abundances of species were tested, and the top 10 most abundant and significantly different species were listed (Figures 4C,D). At the phylum level, the most significant differences were in Actinobacteriota (adjusted *p* = 0.000138), Bacteroidota (adjusted *p* = 0.000166), Desulfobacterota (adjusted *p* = 0.000166), and Sva0485 (adjusted *p* = 0.000138). Bacteria phyla such as Actinobacteriota, Chloroflexi, and Gemmatimonadota were significantly enriched in the rice-wheat model, while Bacteroidota, Desulfobacterota, and Nitrospirota were significantly enriched in the rice-crayfish model. At the genus level, the most abundant and significantly different genera were *Bacteroidetes_vadinHA17* (adjusted *p* = 0.000208), *Sphingomonas* (adjusted *p* = 0.000208), and *Thiobacillus* (adjusted *p* = 0.000277). Bacterial genera such as *KD4-96*, *Sphingomonas*, *Gemmatimonas*, *67–14*, and *Vicinamibacteraceae* were significantly enriched in the rice-wheat model, while *Bacteroidetes_vadinHA17*, *Thiobacillus*, *Anaeromyxobacter*, and *Bathyarchaeia* were significantly enriched in the rice-crayfish model.

The contribution of species at the phylum and genus levels to the differences between the two groups was measured using SIMPER (Similarity Percentages) analysis. The contribution index indicated the degree to which species contribute to the differences between the two groups. A higher contribution index indicated greater differences between the groups for that species. Table 1 shows the species at the phylum level with a contribution index greater than 0.05 and at the genus level with a contribution index greater than 0.01. The results indicated that Actinobacteriota, Bacteroidota, and Desulfobacterota, which were the most significantly different species, also had the highest contribution to the differences in microbial composition between the two groups. At the genus level, *Bacteroidetes_vadinHA17*, *Sphingomonas*, and *Thiobacillus* were the top three annotated species contributing the most to the differences in community composition. Similar to the analysis at the phylum level, these three genera were also the most significantly different bacterial genera.

**Table 1:** SIMPER analysis on phylum and genus levels.

Species		Average abundance		Contribution
		RW	RC	
Phylum	Actinobacteriota	220835.8	70135.74	0.238880
	Bacteroidota	52585.46	137784.2	0.135144
	Desulfobacterota	13877.17	68374.39	0.086455
	Proteobacteria	278073.2	296800.6	0.066861
	Acidobacteriota	118391.9	99793.13	0.065777
	Chloroflexi	85689.25	46614.74	0.063229
	Gemmatimonadota	72367.95	40511.05	0.054626
Genus	*Unclassified*	143977.3	114237.1	0.040651
	*Bacteroidetes_vadinHA17*	405.4593	32101.04	0.033512
	*Uncultured*	155208.1	181815.8	0.032369
	*Sphingomonas*	24576.07	3112.881	0.022693
	*Thiobacillus*	2092.693	23087.2	0.022213
	*SC-I-84*	26899.83	35318.56	0.018306
	*KD4-96*	25267.09	9305.945	0.016975
	*Gemmatimonas*	23784.77	8970.242	0.016721
	*Vicinamibacteraceae*	19959.06	6942.946	0.014624
	*67-14*	17253.82	3880.202	0.014434
	*Bathyarchaeia*	4407.735	15660.32	0.012741
	*Nocardioides*	13872.81	2519.951	0.012136
	*Anaeromyxobacter*	7058.48	17223.3	0.012091
	*Latescibacterota*	8726.095	13288.6	0.011315

Species are the names of species at the phylum and genus levels. Group RW represents soil after wheat planting and group RC represents soil after crayfish farming. The columns RW and RC record the average abundances of the corresponding species in the two groups, respectively. The contribution index indicates the degree to which species contribute to the differences between the two groups. The test was based on Bray–Curtis dissimilarity.

### 3.3 Biomarker screening

Random Forests Prediction Analysis is a classical and efficient machine learning algorithm based on decision trees, belonging to non-linear classifiers. This method was used to screen features that played important roles in grouping as potential biomarkers. The Gini index served as an indicator of the potential of species as biomarkers, with higher values indicating greater importance. According to the Random Forests prediction results (Table 2), at the phylum level, Actinobacteriota, Desulfobacterota, and Spirochaetota were potential biomarkers. At the genus level, *Streptomyces*, *Kribbella*, and *Paludibacter* were potential biomarkers.

**Table 2:** Random forests prediction on phylum and genus levels.

Species		Mean decrease in accuracy	SD decrease in accuracy	Mean decrease in Gini
Phylum	Actinobacteriota	0.024856	0.003310	0.476757
	Desulfobacterota	0.016057	0.002591	0.427958
	Spirochaetota	0.025645	0.003598	0.426069
	Fusobacteriota	0.019824	0.002994	0.382743
	Sva0485	0.019409	0.002886	0.369621
	Bacteroidota	0.012677	0.002055	0.311004
	Chloroflexi	0.010459	0.002031	0.291504
	TA06	0.013758	0.002418	0.283555
	Cloacimonadota	0.009425	0.001835	0.257563
	FCPU426	0.012572	0.002160	0.253015
Genus	*Streptomyces*	0.001071	0.000609	0.040971
	*Kribbella*	0.001883	0.000860	0.040167
	*Paludibacter*	0.001364	0.000569	0.039677
	*Methyloparacoccus*	0.001517	0.000802	0.034537
	*Nannocystis*	0.000571	0.000349	0.033547
	*Dactylosporangium*	0.002294	0.001213	0.033500
	*WCHB1-32*	0.001329	0.000714	0.031917
	*Nocardioides*	0.001413	0.000753	0.031047
	*Actibacter*	0.001417	0.000730	0.031021
	*WCHB1-02*	0.001821	0.000941	0.030401

The table shows the top 10 classified bacterial phyla and genera by importance. Mean decrease in accuracy indicates the contribution of the species to the model’s prediction accuracy. SD decrease in accuracy represents the standard deviation of the species’ importance. Mean decrease in Gini indicates the impact of the species on the heterogeneity of observations at each node in the classification tree. A higher Gini index indicates greater importance of the species.

Similar to the results of differential testing and SIMPER analysis, Actinobacteriota, Desulfobacterota, Bacteroidota, and Chloroflexi were not only significantly different species between the two groups, but also the main species contributing to the differences between the two groups.

### 3.4 Functional prediction analysis

**Figure 6:**
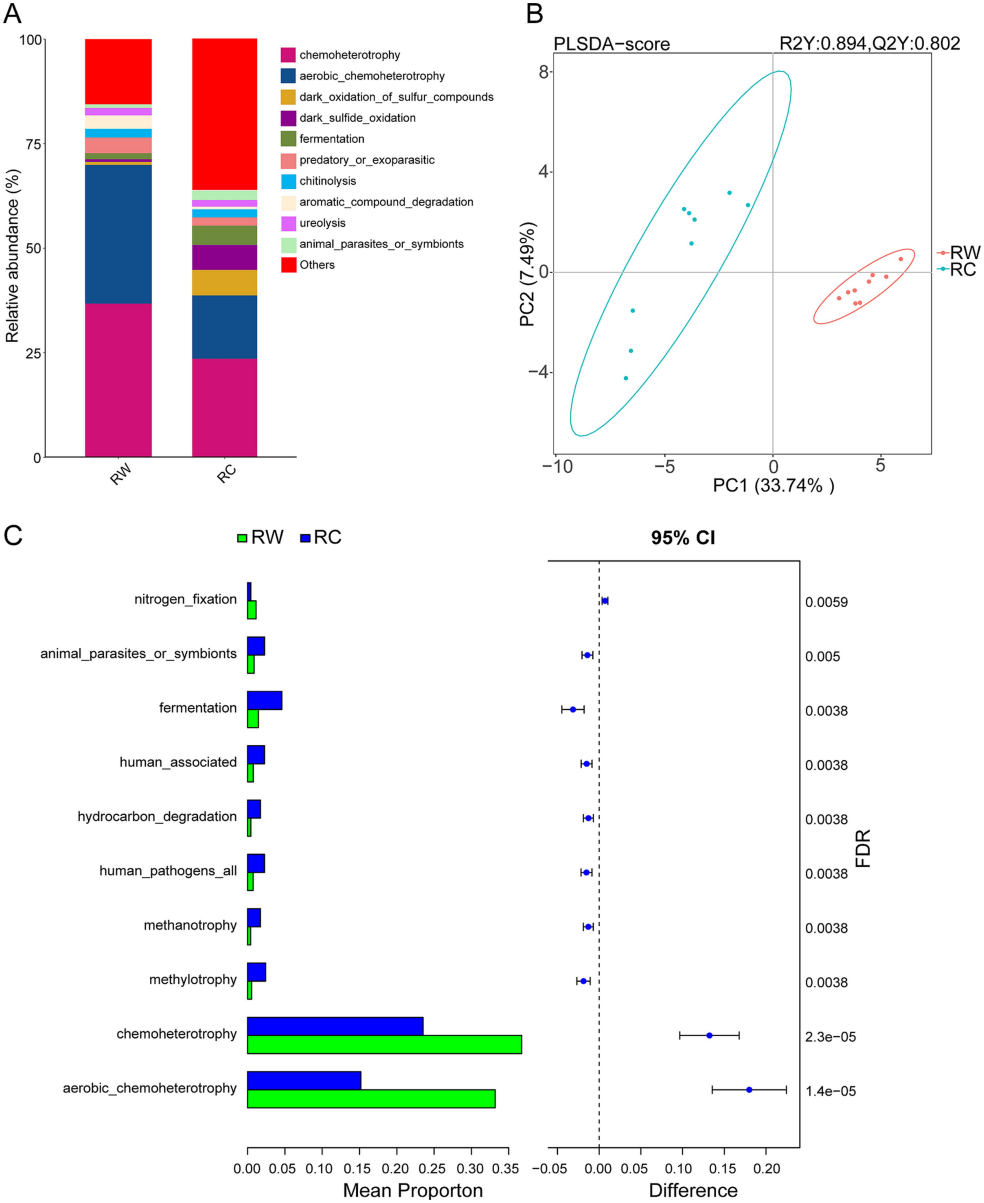
FAPROTAX functional prediction and difference analysis. **(A)** Top 10 predicted functions by abundance in both groups of samples. **(B)** PLS-DA analysis of microbial functional differences between the two groups of samples. **(C)** Difference analysis of functions. Group RW represents soil after wheat planting and group RC represents soil after crayfish farming. FDR indicates the False Discovery Rate.

Ecological functions were analyzed using the FAPROTAX software. The top 10 functional categories by abundance in the samples were chemoheterotrophy, aerobic chemoheterotrophy, dark oxidation of sulfur compounds, dark sulfide oxidation, fermentation, predatory or exoparasitic, chitinolysis, aromatic compound degradation, ureolysis, and animal parasites or symbionts (Figure 6A).

The results of PLS-DA showed that the two groups of samples were clearly separated on axis 1, indicating that different land use patterns significantly alter soil microbial functions (Figure 6B). Compared to rice-wheat model, soil microbial functions in crayfish-raised plots were more dispersed on the coordinate axis, with more functions classified as “others,” suggesting greater functional diversity in the microbial community.

Significant differential functional categories (*p* < 0.05) obtained using the Kruskal–Wallis test were clustered and a corresponding heatmap was created (Supplementary Figure S1). According to the clustering results, the predicted functions were divided into two clusters. Most functions were positively correlated with group RC, and a few were positively correlated with group RW. This result was similar to the differential species.

Bar charts of the top 10 most different functional categories were plotted and analyzed (Figure 6C). The differential analysis results showed that aerobic chemoheterotrophy and chemoheterotrophy were the two most significantly different functions. The relative abundances of these two functions in rice-wheat plots were significantly higher than in rice-crayfish plots. In contrast, the relative abundances of methylotrophy, human pathogens all, methanotrophy, hydrocarbon degradation, and human associated functions in rice-crayfish plots were significantly higher than in rice-wheat plots.

### 3.5 Co-occurrence network analysis

**Figure 7:**
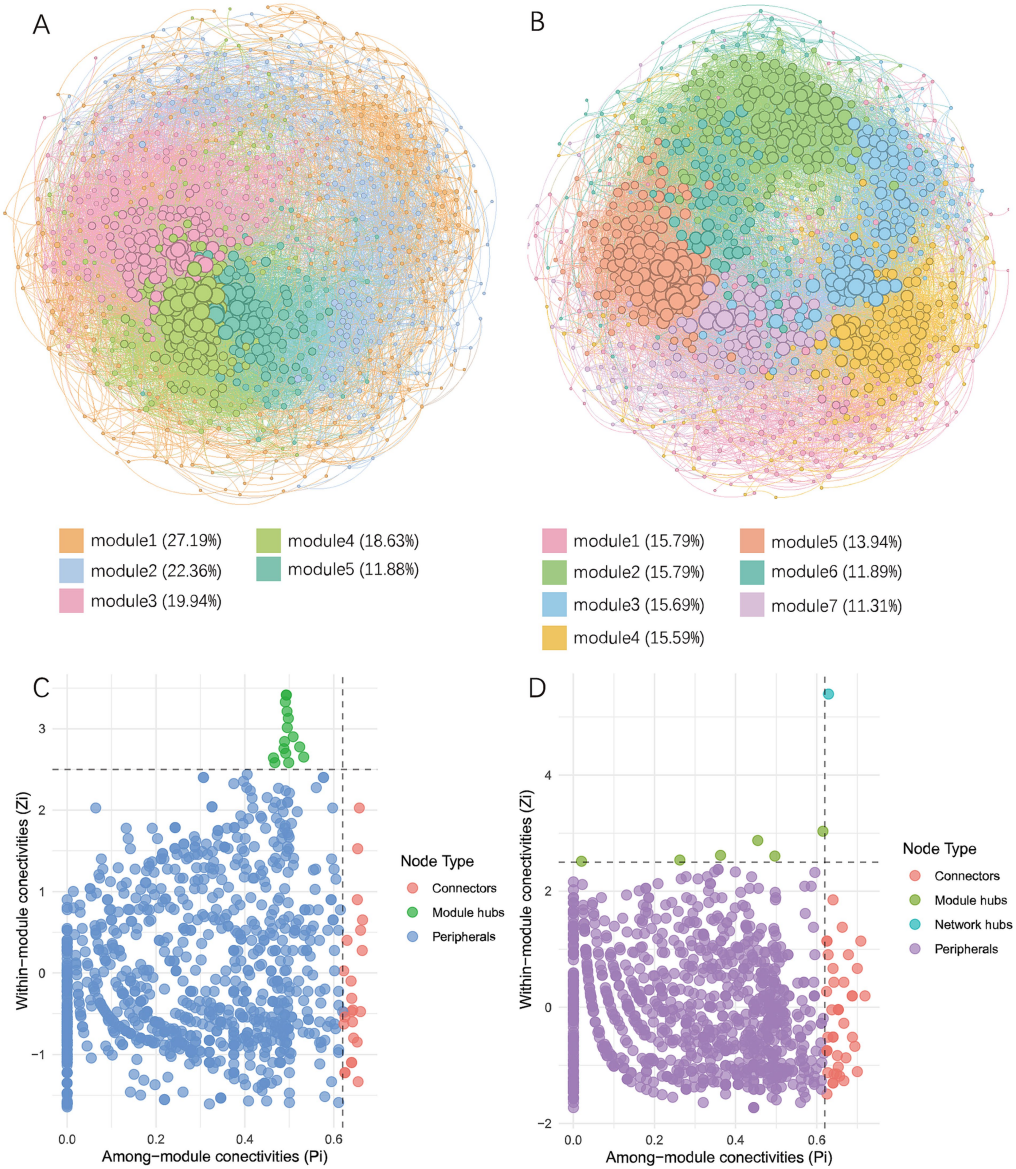
Co-occurrence network analysis of soil microorganisms. **(A)** Co-occurrence network of soil microbes in the wheat group. **(B)** Co-occurrence network of soil microbes in the crayfish group. Different colors represent corresponding modules and their proportions. **(C)** Topological properties of nodes in the co-occurrence network of the wheat group. **(D)** Topological properties of nodes in the co-occurrence network of the crayfish group. Group RW represents soil after wheat planting and group RC represents soil after crayfish farming.

Co-occurrence networks of bacterial communities in the soils were constructed to explore interactions between different species (Figure 7; Table 3). The results showed that in wheat-planted soil, the number of nodes was 993, lower than the 1,026 in crayfish-raising soil, but the network had more edges (24,155), a higher average degree (48.65), a higher average clustering coefficient (0.452), and a lower average path distance (3.144). This indicated that the microbial community in wheat soil has more internal connections than external connections. The crayfish group had a higher modularity (0.549), indicating a higher degree of modularity. The positive correlation ratio in the wheat group was 58.00%, with the remaining 42.00% being negative correlation. The crayfish group had a stronger positive correlation, at 60.06%.

**Table 3:** Topological indicators of co-occurrence networks.

Topological indicators	RW	RC
Nodes	993	1,026
Edges	24,155	18,658
Average degree	48.65	36.37
Average clustering coefficient	0.452	0.435
Average path distance	3.144	3.213
Modularity	0.373	0.549
Positive correlation (%)	58.00	60.06
Negative correlation (%)	42.00	39.94

Group RW represents soil after wheat planting and group B represents soil after crayfish farming.

Nodes with important roles were identified based on their *Zi* and *Pi* values (Supplementary Table S2). In wheat-plating soil, there were 24 nodes acting as connectors and 15 nodes as module hubs. In crayfish-raising soil, there were 40 nodes acting as connectors, 6 nodes as module hubs, and one node with the attribute of a network hub. This showed that the microbial network in crayfish soil was more connected and stable. The annotation results showed that in the rice-wheat rotation mode, connectors were mainly composed of Proteobacteria (12) and Actinobacteriota (6), and module hubs were mainly composed of Proteobacteria (6), Acidobacteriota (4), and Actinobacteriota (3). In the rice-crayfish rotation mode, connectors were mainly derived from Proteobacteria (12), Actinobacteriota (7), Bacteroidota (5), Verrucomicrobiota (5), and Chloroflexi (4). Module hubs were mainly derived from Desulfobacterota (2) and Proteobacteria (2). The network hub node belonged to the Nitrospirota phylum and the *4-29-1* genus.

## 4 Discussion

Environmental heterogeneity significantly influences the distribution of microorganisms, leading to differences in their niches and functions (Xu et al., 2024). Our study found that different crop rotation methods in integrated rice farming significantly altered the soil microbial community, driving significant differences and affecting their functions.

### 4.1 Different agricultural patterns drive differential enrichment of soil microorganisms

In terms of species composition, this experiment found that wheat planting effectively increased the abundance of Actinobacteriota in the soil, while crayfish farming increased the abundance of Bacteroidota and Desulfobacterota. These three bacterial phyla, Actinobacteriota, Bacteroidota, and Desulfobacterota, were the main species causing differences in microbial composition between the two groups.

Acidobacteriota is a generally aerobic, slow-growing, and oligotrophic taxa. Actinobacteriota is widely present in soil and can exhibit strong adaptability in salt tolerance (Wang et al., 2024b), and drought resistance, even becoming keystone organisms in microbial networks (Pan et al., 2024). The Bacteroidota and Desulfobacterota phyla are anaerobic and primarily use substances in the environment to produce energy. Bacteroidota is widely found in sediments of aquaculture (Wang et al., 2022). This phylum is generally anaerobic, fast-growing, and copiotrophic in soils or sediments (Lin and Lin, 2022). Bacteroidota has the ability to secrete various carbohydrate-active enzymes (CAZymes) to degrade complex carbohydrate in soil (Larsbrink and McKee, 2020). Desulfobacterota perform strictly anaerobic chemoorganotrophic, chemolithoheterotrophic or chemolithoautotrophic growth by respiratory or fermentative metabolism (Wang et al., 2022).

At the genus level, *Bacteroidetes_vadinHA17*, *Sphingomonas*, and *Thiobacillus* contributed the most to the differences. *Sphingomonas* was enriched in the rice-wheat pattern, while *Bacteroidetes_vadinHA17* and *Thiobacillus* were enriched in the rice-crayfish pattern. *Sphingomonas* is usually enriched in agricultural soil (Alahmad et al., 2018), helps plants acquire nutrients (such as phosphorus, iron) and improves plant biomass production (Mazoyon et al., 2023). *Bacteroidetes_vadinHA17* is an anaerobic or facultative bacterium (Li et al., 2024), could degrade glucose into small molecules such as acetic acid, propionic acid and H_2_/CO_2_ (Wang R. et al., 2021). *Thiobacillus* is a sulfur-oxidizing bacterium that plays an important role in the sulfur cycle, primarily performing denitrification (Skinner and Jahren, 2007; Zhao et al., 2024). As an autotrophic bacterium, it can oxidize inorganic sulfur compounds such as H_2_S to obtain energy and produce sulfate. In agriculture, it is considered to increase the availability of sulfur, providing nutrients for crops (Gopalakrishnan et al., 2020).

Depending on the different soil environments, the two groups of microorganisms exhibited extremely different environmental selectivity and lifestyles. The analysis results identified Actinobacteriota, Desulfobacterota, and Spirochaetota as potential biomarkers. Spirochaetota can decompose cellulose and are widely present in the digestive tracts of herbivorous animals (Dhakal et al., 2024; Salgado et al., 2024). This may be related to the rich vegetation in the rice-wheat rotation pattern. Crayfish farming ponds are artificial aquatic ecosystems characterized by long-term flooding, closure, and lack of vegetation. The long-term flooding during farming usually reduces oxygen levels, creating an anoxic environment that is conducive to anaerobic life for microorganisms in the sediments (Wang M. et al., 2024). This indicates that different rotation patterns drive the soil microbial community to enrich in two different directions, with Actinobacteriota and Desulfobacterota possibly being representative species.

### 4.2 Different crop rotation patterns significantly altered the metabolic pathways of soil microorganisms

Different land use patterns not only significantly changed the types and abundances of soil microorganisms but also altered their metabolic pathways. FAPROTAX analysis indicated that the microbial functions in both patterns were mostly related to carbon cycling. The bacterial functions in the rice-wheat pattern were concentrated on aerobic chemoheterotrophy and chemoheterotrophy, indicating that soil microorganisms primarily obtain carbon and energy by oxidizing organic matter, with an active chemical degradation process (Wu et al., 2024). These metabolic pathways are widely present in dryland farming patterns with frequent oxygen exchange and rich nutrients (Wang et al., 2024a).

Compared to the rice-wheat pattern with more frequent oxygen exchange, the microorganisms in the low-oxygen rice-crayfish pattern had more complex and diverse metabolic pathways, utilized various methods to obtain nutrients. In addition to aerobic chemoheterotrophy and chemoheterotrophy, the main functions also included methylotrophy, hydrocarbon degradation, and methanotrophy. Researchers (Zhang et al., 2024) demonstrated that flooding decreased the stability of dissolved organic carbon in surface soil and enhanced the simple depolymerization of its structure. Consequently, the integrated rice-crayfish system enriches the substrate availability for microbial metabolism in surface soil, promoting the decomposition of soil humus components while simultaneously suppressing polysaccharide degradation. This mechanism ultimately enhances the accumulation of organic carbon in deeper soil layers. It was proved that this integrated model has greater carbohydrate metabolism ability when compared with non- integrated models (Zhu et al., 2023). In our research, the predicted metabolism pathways indicate that microorganisms in rice-crayfish model have the ability to obtain carbon and energy from organic substrates containing no carbon–carbon bonds (C1 compounds, such as methane, methanol, halogenated methane, etc.) (Chistoserdova, 2011). Methylotrophy and methanotrophy are very significant nutrient utilization methods in aquaculture sediments (Huang et al., 2024). In the low-oxygen environment of sediments, methanotrophs can utilize limited oxygen for metabolism and produce substances such as methanol and formaldehyde to supply other microorganisms, forming a food chain (Chistoserdova and Kalyuzhnaya, 2018). This indicates that the nutrient flow in the crayfish farming environment is mainly driven by carbon cycling, and these metabolic pathways are often related to carbon fixation. Therefore, the rice-crayfish rotation pattern has the potential to reduce greenhouse gas emissions (Wang L. et al., 2024).

### 4.3 Different crop rotation patterns altered the structure of soil microbial networks

Key species in the network were selected based on the *Zi* and *Pi* values of the nodes. In both crop rotation patterns, species belonging to Proteobacteria and Actinobacteriota simultaneously acted as connectors and module hubs, indicating that these two phyla are important components in constructing soil microbial networks. Similar to existing research (Zhu et al., 2022), this experiment also found that raising crayfish in agricultural systems helped build positive and stable environmental microbial networks. Compared to planting wheat during rotation, raising crayfish can effectively increase the number and diversity of microorganisms in the soil, enhance positive correlations in the microbial network, and thereby increase resistance to environmental stress. In the soil of the rice-crayfish pattern, the microbial network had a higher degree of modularity, and a more diverse range of species (such as Bacteroidota, Verrucomicrobiota, Desulfobacterota, etc.) were important nodes in the microbial network. It is speculated that the diverse microorganisms in this pattern occupy different niches in the ecological network and perform various functions. Additionally, there was a node with the attribute of network hubs, which belongs to the phylum Nitrospirota and the genus *4-29-1*. There is currently very little research on *4-29-1* (Zhou et al., 2024), but it is inferred from the phylum level that this species has the ability to metabolize nitrogen-containing organic compounds (Guo et al., 2024).

Previous studies have mostly focused on the microbial communities in rice-crayfish co-cultivation patterns or compared rice-crayfish rotation with single rice planting (Jiang and Cao, 2021; Wei et al., 2021; Zhu et al., 2022; Huang et al., 2023). These studies have proven that raising crayfish in rice paddies helps increase biodiversity and resource utilization efficiency. If only rice or other crops are planted as producers in the field, it can lead to competition for energy from weeds or phytoplankton, or excessive waste of photosynthetic products due to the lack of consumers. Long-term maintenance of a single land use pattern may reduce the content of beneficial soil microorganisms and increase the abundance of pathogenic bacteria (Liu et al., 2021). Similar to the results of these studies, this experiment also found that compared to planting wheat, raising crayfish can effectively increase the richness and diversity of soil microorganisms and improve the structure of soil bacteria. Rich rhizosphere microorganisms are beneficial for crop growth, increasing productivity, and playing important roles in maintaining nutrient absorption and biological defense (Jaiswal et al., 2022). In the rice-crayfish rotation system, raising crayfish and increasing the richness and diversity of soil microorganisms may be beneficial for the subsequent growth of rice, forming a virtuous cycle system.

A diversified crop planting system can effectively enhance agricultural productivity and economic benefits, strengthen farming resilience, and improve adaptability to environmental changes (Yang et al., 2024). Positive network correlations and a large number of connected species imply extensive cooperative relationships among microorganisms, including cross-feeding, syntrophism, mutualism, and commensalism (Wang Q. et al., 2024). Therefore, constructing a symbiotic system of crayfish-rice-soil-microorganisms can help achieve resource conservation and ecological balance (Jiang and Cao, 2021).

## 5 Conclusion

This study compared the effects of different rotation methods (wheat planting and crayfish farming) on soil microbial communities and functions. The results indicated that different rotation models in integrated rice farming systems can significantly alter soil microbial community composition, driving significant differences and affecting their functions. Compared to wheat planting, crayfish farming can effectively increase the number and diversity of soil microorganisms. The identified differential microorganisms and potential biomarkers can serve as key species to distinguish the soil in two farming groups. This study provides theoretical data for integrated rice farming systems and further optimizes land management practices to improve soil environments. It is worthy to further explore the impact of these two rotation patterns on the physicochemical properties and material composition of soil, along with the relationships between these indicators and microorganisms.

## Data Availability

The datasets presented in this study can be found in online repositories. The names of the repository/repositories and accession number(s) can be found at: https://www.ncbi.nlm.nih.gov/, PRJNA1185463.

## Footnotes

^1^ http://www.arb-silva.de
