# Supplementary material for: Cultivating crayfish (Procambarus clarkii) significantly enhances the quantity and diversity of soil microorganisms: evidence from the comparison of rice-wheat and rice-crayfish rotation models
Source: Front Microbiol. 2025 Feb 3;16:1528883. doi: 10.3389/fmicb.2025.1528883 (PMC11830727; doi:10.3389/fmicb.2025.1528883)
Supplement: Supplementary file 1 [file Data_Sheet_1.docx]

Supplementary Material

# Supplementary Figures and Tables

## Supplementary Figures

**
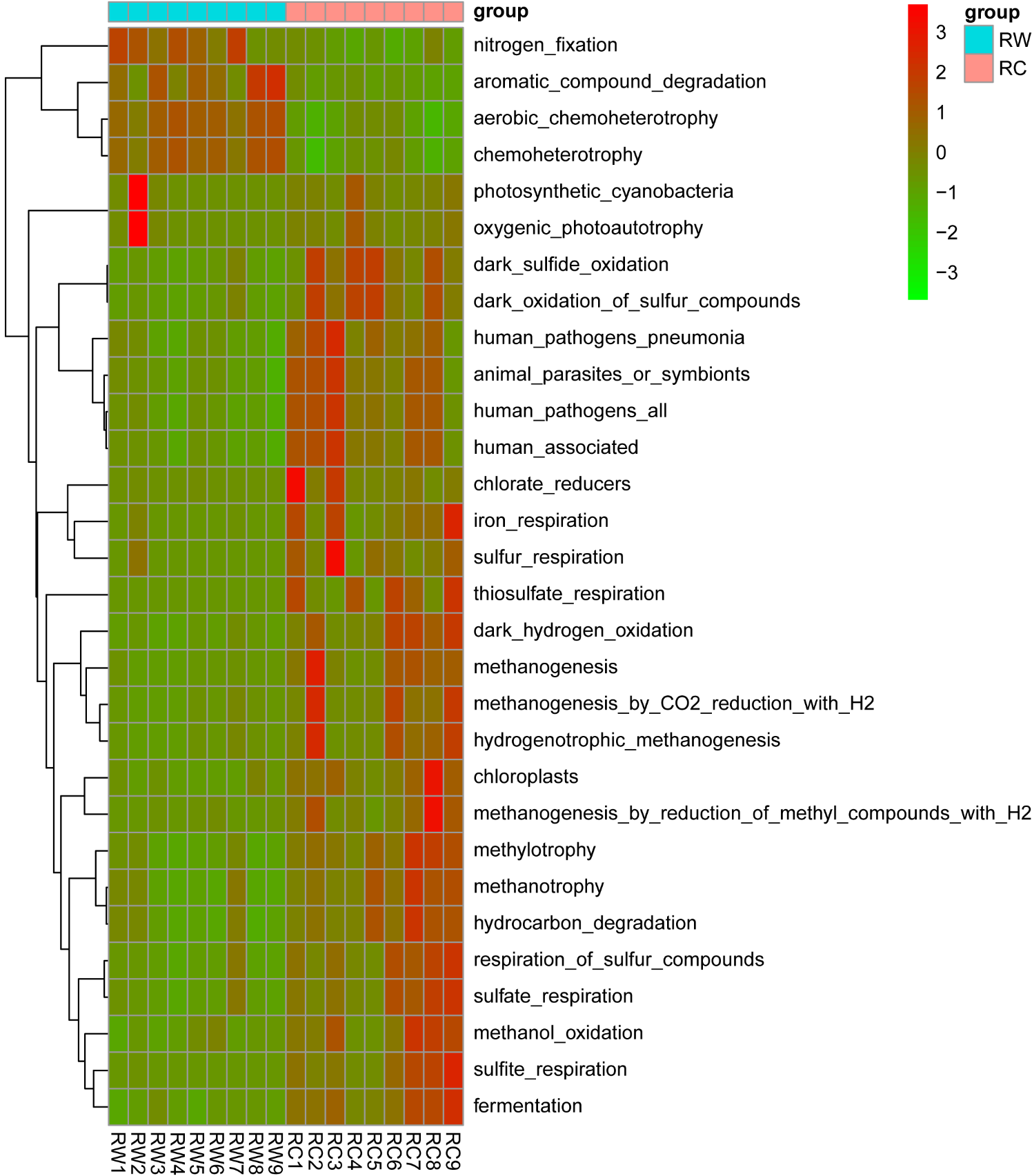
**

**Supplementary Figure 1.** Clustering heatmap of the top 50 differential functions predicted by FAPROTAX

Group RW represents soil after wheat planting and group RC represents soil after crayfish farming.

## Supplementary Figures

Table S1 Statistics of sequencing and normalized data for samples

| Sample | Raw reads | Filtered reads | Denoised reads | Merged reads | ASVs  number | EvenSeqs  number | EvenASVs  number |
| --- | --- | --- | --- | --- | --- | --- | --- |
| RW1 | 100386 | 99819 | 96184 | 88185 | 2,722 | 50971 | 2,682 |
| RW2 | 94388 | 93767 | 89346 | 79855 | 3,145 | 50971 | 3,130 |
| RW3 | 95288 | 94738 | 89944 | 77880 | 2,934 | 50971 | 2,922 |
| RW4 | 88375 | 87912 | 84397 | 75887 | 2,575 | 50971 | 2,570 |
| RW5 | 87981 | 87543 | 83870 | 77180 | 2,625 | 50971 | 2,607 |
| RW6 | 90117 | 89592 | 85786 | 77265 | 2,825 | 50971 | 2,804 |
| RW7 | 89574 | 89026 | 84946 | 76267 | 2,834 | 50971 | 2,818 |
| RW8 | 89533 | 88810 | 85222 | 78398 | 2,325 | 50971 | 2,314 |
| RW9 | 87515 | 86892 | 83743 | 76153 | 2,084 | 50971 | 2,075 |
| RC1 | 89145 | 88497 | 83317 | 74536 | 3,142 | 50971 | 3,123 |
| RC2 | 89373 | 88795 | 83708 | 74485 | 2,968 | 50971 | 2,953 |
| RC3 | 89152 | 88437 | 83319 | 75027 | 3,159 | 50971 | 3,143 |
| RC4 | 90906 | 90533 | 87175 | 81651 | 2,709 | 50971 | 2,686 |
| RC5 | 89536 | 88898 | 84681 | 77290 | 2,782 | 50971 | 2,762 |
| RC6 | 95558 | 94929 | 89881 | 80575 | 3,025 | 50971 | 2,996 |
| RC7 | 88682 | 88310 | 83178 | 73411 | 3,143 | 50971 | 3,123 |
| RC8 | 90534 | 90221 | 85655 | 78238 | 3,006 | 50971 | 2,983 |
| RC9 | 93227 | 92715 | 88053 | 80710 | 3,008 | 50971 | 2,986 |

Sample ID represents sample name. Raw read represents raw data volume. Filtered read represents data volume after removing low-quality sequences. Denoised read represents denoised sequence data volume, namely effective sequence volume. Merged read represents sequence volume after merging. Non-chimeric read represents sequence volume after removing chimeras, namely high-quality sequence volume. Non-singleton read represents sequence volume after removing singletons. ASV number represents number of ASV sequences for each sample. Even reads number represents Sequence data volume for each sample after rarefaction. Even ASV number represents number of ASV sequences for each sample after rarefaction. Group RW represents soil after wheat planting and group RC represents soil after crayfish farming.

Table S2 The details of nodes in the co-occurrence network from wheat-rice and crayfish-rice models

| Group | Node types | *Zi* | *Pi* | Kingdom | Phylum | Class | Order | Family | Genus |
| --- | --- | --- | --- | --- | --- | --- | --- | --- | --- |
| RW | Connectors | 2.0267 | 0.6578 | Bacteria | Acidobacteriota | Vicinamibacteria | Vicinamibacterales | uncultured | *uncultured* |
|  |  | 1.5265 | 0.6538 | Bacteria | Proteobacteria | Gammaproteobacteria | Xanthomonadales | Rhodanobacteraceae | *Rhodanobacter* |
|  |  | 0.9012 | 0.6531 | Bacteria | Proteobacteria | Gammaproteobacteria | Burkholderiales | SC-I-84 | *SC-I-84* |
|  |  | 0.6511 | 0.6648 | Bacteria | Gemmatimonadota | Gemmatimonadetes | Gemmatimonadales | Gemmatimonadaceae | *uncultured* |
|  |  | 0.5261 | 0.6605 | Bacteria | Proteobacteria | Alphaproteobacteria | Elsterales | uncultured | *uncultured* |
|  |  | 0.4010 | 0.6298 | Bacteria | Proteobacteria | Alphaproteobacteria | Acetobacterales | Acetobacteraceae | *uncultured* |
|  |  | 0.2760 | 0.6641 | Bacteria | Proteobacteria | Alphaproteobacteria | Rhizobiales | Xanthobacteraceae | *uncultured* |
|  |  | 0.0259 | 0.6224 | Bacteria | Proteobacteria | Alphaproteobacteria | Elsterales | uncultured | *uncultured* |
|  |  | -0.0992 | 0.6391 | Bacteria | Actinobacteriota | Actinobacteria | Frankiales | Sporichthyaceae | *uncultured* |
|  |  | -0.3109 | 0.6403 | Bacteria | Proteobacteria | Alphaproteobacteria | Rhizobiales | Xanthobacteraceae | *Pseudolabrys* |
|  |  | -0.4542 | 0.6427 | Bacteria | Proteobacteria | Gammaproteobacteria | Gammaproteobacteria_Incertae_Sedis | Unknown_Family | *Acidibacter* |
|  |  | -0.4743 | 0.6400 | Bacteria | Actinobacteriota | Thermoleophilia | Solirubrobacterales | 67-14 | *67-14* |
|  |  | -0.4743 | 0.6600 | Bacteria | Chloroflexi | TK10 | TK10 | TK10 | *TK10* |
|  |  | -0.4829 | 0.6240 | Bacteria | Actinobacteriota | Thermoleophilia | Gaiellales | Gaiellaceae | *Gaiella* |
|  |  | -0.5402 | 0.6250 | Bacteria | Armatimonadota | Chthonomonadetes | Chthonomonadales | Chthonomonadaceae | *Chthonomonas* |
|  |  | -0.5994 | 0.6420 | Bacteria | Actinobacteriota | Thermoleophilia | Gaiellales | Gaiellaceae | *Gaiella* |
|  |  | -0.6262 | 0.6228 | Bacteria | Proteobacteria | Alphaproteobacteria | Reyranellales | Reyranellaceae | *Reyranella* |
|  |  | -0.7982 | 0.6446 | Bacteria | Bacteroidota | Bacteroidia | Chitinophagales | Chitinophagaceae | *Flavisolibacter* |
|  |  | -0.8495 | 0.6531 | Bacteria | Actinobacteriota | Actinobacteria | Micromonosporales | Micromonosporaceae | *-* |
|  |  | -1.0996 | 0.6400 | Bacteria | Actinobacteriota | Thermoleophilia | Solirubrobacterales | Solirubrobacteraceae | *Solirubrobacter* |
|  |  | -1.0996 | 0.6400 | Bacteria | Proteobacteria | Gammaproteobacteria | Xanthomonadales | Rhodanobacteraceae | *Luteibacter* |
|  |  | -1.2246 | 0.6250 | Bacteria | Nitrospirota | Nitrospiria | Nitrospirales | Nitrospiraceae | *Nitrospira* |
|  |  | -1.2246 | 0.6250 | Bacteria | Proteobacteria | Gammaproteobacteria | Burkholderiales | Nitrosomonadaceae | *Nitrosomonas* |
|  |  | -1.3349 | 0.6550 | Bacteria | Proteobacteria | Gammaproteobacteria | Burkholderiales | SC-I-84 | *SC-I-84* |
|  | Module hubs | 3.4155 | 0.4935 | Bacteria | Proteobacteria | Alphaproteobacteria | Sphingomonadales | Sphingomonadaceae | *-* |
|  |  | 3.4155 | 0.4935 | Bacteria | Acidobacteriota | Vicinamibacteria | Vicinamibacterales | uncultured | *uncultured* |
|  |  | 3.3295 | 0.4908 | Bacteria | Proteobacteria | Alphaproteobacteria | Sphingomonadales | Sphingomonadaceae | *Sphingomonas* |
|  |  | 3.2148 | 0.4951 | Bacteria | Gemmatimonadota | Gemmatimonadetes | Gemmatimonadales | Gemmatimonadaceae | *uncultured* |
|  |  | 3.1288 | 0.4977 | Bacteria | Actinobacteriota | Actinobacteria | Propionibacteriales | Nocardioidaceae | *-* |
|  |  | 3.0142 | 0.4953 | Bacteria | Actinobacteriota | Actinobacteria | Micrococcales | Intrasporangiaceae | *-* |
|  |  | 2.9020 | 0.5084 | Bacteria | Proteobacteria | Alphaproteobacteria | Caulobacterales | Caulobacteraceae | *Phenylobacterium* |
|  |  | 2.8422 | 0.4895 | Bacteria | Proteobacteria | Alphaproteobacteria | Sphingomonadales | Sphingomonadaceae | *-* |
|  |  | 2.7769 | 0.5231 | Bacteria | Acidobacteriota | Subgroup_25 | Subgroup_25 | Subgroup_25 | *Subgroup_25* |
|  |  | 2.7562 | 0.4879 | Bacteria | Proteobacteria | Alphaproteobacteria | Sphingomonadales | Sphingomonadaceae | *Sphingomonas* |
|  |  | 2.6989 | 0.4918 | Bacteria | Acidobacteriota | Vicinamibacteria | Vicinamibacterales | Vicinamibacteraceae | *Vicinamibacteraceae* |
|  |  | 2.6519 | 0.5322 | Bacteria | Acidobacteriota | Holophagae | Subgroup_7 | Subgroup_7 | *Subgroup_7* |
|  |  | 2.6415 | 0.4643 | Bacteria | Actinobacteriota | Thermoleophilia | Solirubrobacterales | Solirubrobacteraceae | *Conexibacter* |
|  |  | 2.5842 | 0.4985 | Bacteria | Bacteroidota | Bacteroidia | Chitinophagales | Chitinophagaceae | *Flavisolibacter* |
|  |  | 2.5842 | 0.4673 | Bacteria | Proteobacteria | Alphaproteobacteria | Micropepsales | Micropepsaceae | *Micropepsis* |
| RC | Connectors | 1.8509 | 0.6400 | Bacteria | Actinobacteriota | Coriobacteriia | OPB41 | OPB41 | *OPB41* |
|  |  | 1.3784 | 0.6627 | Bacteria | Chloroflexi | KD4-96 | KD4-96 | KD4-96 | *KD4-96* |
|  |  | 1.1422 | 0.6250 | Bacteria | Verrucomicrobiota | Verrucomicrobiae | Pedosphaerales | Pedosphaeraceae | *ADurb.Bin063-1* |
|  |  | 1.1422 | 0.6250 | Archaea | Thermoplasmatota | Thermoplasmata | Methanomassiliicoccales | Methanomassiliicoccaceae | *uncultured* |
|  |  | 1.1422 | 0.7083 | Bacteria | Chloroflexi | Chloroflexia | Thermomicrobiales | JG30-KF-CM45 | *JG30-KF-CM45* |
|  |  | 0.9059 | 0.6777 | Bacteria | Latescibacterota | Latescibacterota | Latescibacterota | Latescibacterota | *Latescibacterota* |
|  |  | 0.9059 | 0.6281 | Bacteria | Verrucomicrobiota | Verrucomicrobiae | Pedosphaerales | Pedosphaeraceae | *-* |
|  |  | 0.6697 | 0.6400 | Archaea | Crenarchaeota | Bathyarchaeia | Bathyarchaeia | Bathyarchaeia | *Bathyarchaeia* |
|  |  | 0.6697 | 0.7000 | Bacteria | Firmicutes | Clostridia | - | - | *-* |
|  |  | 0.4334 | 0.6420 | Archaea | Crenarchaeota | Bathyarchaeia | Bathyarchaeia | Bathyarchaeia | *Bathyarchaeia* |
|  |  | 0.4334 | 0.6667 | Bacteria | Proteobacteria | Gammaproteobacteria | Burkholderiales | Comamonadaceae | *Rhizobacter* |
|  |  | 0.2732 | 0.6242 | Bacteria | Verrucomicrobiota | Verrucomicrobiae | Pedosphaerales | Pedosphaeraceae | *ADurb.Bin063-1* |
|  |  | 0.1972 | 0.7188 | Bacteria | Proteobacteria | Alphaproteobacteria | Sphingomonadales | Sphingomonadaceae | *Sphingomonas* |
|  |  | 0.1972 | 0.6875 | Bacteria | Proteobacteria | Alphaproteobacteria | Rhizobiales | Rhizobiales_Incertae_Sedis | *uncultured* |
|  |  | 0.1972 | 0.6875 | Bacteria | Proteobacteria | Gammaproteobacteria | Burkholderiales | Methylophilaceae | *-* |
|  |  | -0.0391 | 0.6531 | Bacteria | Bacteroidota | Bacteroidia | Chitinophagales | Saprospiraceae | *uncultured* |
|  |  | -0.0391 | 0.6531 | Bacteria | Verrucomicrobiota | Verrucomicrobiae | Pedosphaerales | Pedosphaeraceae | *ADurb.Bin063-1* |
|  |  | -0.0391 | 0.6531 | Bacteria | Chloroflexi | Anaerolineae | SJA-15 | SJA-15 | *SJA-15* |
|  |  | -0.0422 | 0.6370 | Bacteria | Gemmatimonadota | S0134_terrestrial_group | S0134_terrestrial_group | S0134_terrestrial_group | *S0134_terrestrial_group* |
|  |  | -0.2753 | 0.6667 | Bacteria | Desulfobacterota | Syntrophorhabdia | Syntrophorhabdales | Syntrophorhabdaceae | *Syntrophorhabdus* |
|  |  | -0.5116 | 0.6400 | Bacteria | Proteobacteria | Gammaproteobacteria | Burkholderiales | Nitrosomonadaceae | *Ellin6067* |
|  |  | -0.5116 | 0.6400 | Bacteria | Proteobacteria | Gammaproteobacteria | Burkholderiales | Oxalobacteraceae | *Massilia* |
|  |  | -0.5154 | 0.6272 | Bacteria | Proteobacteria | Gammaproteobacteria | Burkholderiales | SC-I-84 | *SC-I-84* |
|  |  | -0.5154 | 0.6880 | Bacteria | Bacteroidota | Bacteroidia | Chitinophagales | Chitinophagaceae | *Aurantisolimonas* |
|  |  | -0.6731 | 0.6939 | Bacteria | Proteobacteria | Gammaproteobacteria | Steroidobacterales | Steroidobacteraceae | *uncultured* |
|  |  | -0.7478 | 0.6250 | Bacteria | Acidobacteriota | Holophagae | Subgroup_7 | Subgroup_7 | *Subgroup_7* |
|  |  | -0.7478 | 0.6250 | Bacteria | Verrucomicrobiota | Verrucomicrobiae | Pedosphaerales | Pedosphaeraceae | *ADurb.Bin063-1* |
|  |  | -0.7478 | 0.6250 | Bacteria | Bacteroidota | Bacteroidia | Bacteroidales | Bacteroidetes_vadinHA17 | *Bacteroidetes_vadinHA17* |
|  |  | -0.8309 | 0.6505 | Bacteria | Bacteroidota | Bacteroidia | Bacteroidetes_VC2.1_Bac22 | Bacteroidetes_VC2.1_Bac22 | *Bacteroidetes_VC2.1_Bac22* |
|  |  | -0.8703 | 0.6719 | Bacteria | Actinobacteriota | Actinobacteria | PeM15 | PeM15 | *PeM15* |
|  |  | -1.0280 | 0.6528 | Bacteria | Actinobacteriota | Actinobacteria | Micrococcales | Intrasporangiaceae | *-* |
|  |  | -1.1069 | 0.7000 | Bacteria | Proteobacteria | Gammaproteobacteria | Burkholderiales | Hydrogenophilaceae | *Thiobacillus* |
|  |  | -1.1255 | 0.6272 | Bacteria | Actinobacteriota | Actinobacteria | Propionibacteriales | Nocardioidaceae | *-* |
|  |  | -1.1463 | 0.6420 | Bacteria | Bacteroidota | Ignavibacteria | Ignavibacteriales | UA-50 | *UA-50* |
|  |  | -1.1474 | 0.6484 | Bacteria | Acidobacteriota | Vicinamibacteria | Subgroup_17 | Subgroup_17 | *Subgroup_17* |
|  |  | -1.1857 | 0.6563 | Bacteria | Proteobacteria | Gammaproteobacteria | Steroidobacterales | Steroidobacteraceae | *uncultured* |
|  |  | -1.2646 | 0.6667 | Bacteria | Acidobacteriota | Holophagae | Subgroup_7 | Subgroup_7 | *Subgroup_7* |
|  |  | -1.3040 | 0.6400 | Bacteria | Proteobacteria | Gammaproteobacteria | Steroidobacterales | Steroidobacteraceae | *uncultured* |
|  |  | -1.3040 | 0.6400 | Bacteria | Proteobacteria | Gammaproteobacteria | KI89A_clade | KI89A_clade | *KI89A_clade* |
|  |  | -1.4916 | 0.6250 | Bacteria | Chloroflexi | OLB14 | OLB14 | OLB14 | *OLB14* |
|  | Module hubs | 3.0322 | 0.6150 | Bacteria | Bacteroidota | Bacteroidia | Bacteroidales | Bacteroidetes_BD2-2 | *Bacteroidetes_BD2-2* |
|  |  | 2.8735 | 0.4544 | Bacteria | Desulfobacterota | Desulfovibrionia | Desulfovibrionales | Desulfomicrobiaceae | *Desulfomicrobium* |
|  |  | 2.6178 | 0.3628 | Bacteria | Proteobacteria | Gammaproteobacteria | Burkholderiales | B1-7BS | *B1-7BS* |
|  |  | 2.6055 | 0.4968 | Bacteria | Desulfobacterota | Desulfuromonadia | Geobacterales | Geobacteraceae | *uncultured* |
|  |  | 2.5364 | 0.2624 | Bacteria | Proteobacteria | Gammaproteobacteria | Burkholderiales | Gallionellaceae | *Sideroxydans* |
|  |  | 2.5161 | 0.0202 | Bacteria | Firmicutes | Bacilli | Izemoplasmatales | Izemoplasmataceae | *Izemoplasmataceae* |
|  | Network hubs | 5.3946 | 0.6289 | Bacteria | Nitrospirota | 4-29-1 | 4-29-1 | 4-29-1 | *4-29-1* |

Group RW represents soil after wheat planting and group RC represents soil after crayfish farming.
